# Drug Utilization and Potential Drug-Drug Interactions within an Intensive Care Unit at a University Tertiary Care Hospital in Egypt

**DOI:** 10.3390/pharmacy10040096

**Published:** 2022-08-07

**Authors:** Dima F. Obeid, Adel H. Karara

**Affiliations:** 1 Clinical Pharmacy Unit, Internal Medicine Hospital, Ain-Shams University Hospital, Cairo 11591, Egypt; 2 Department of Pharmaceutical Sciences, School of Pharmacy, University of Maryland Eastern Shore, Princess Anne, MD 21853, USA

**Keywords:** drug utilization, drug-drug interactions, intensive care unit, medication safety

## Abstract

There are few reports on drug utilization and drug-drug interactions in Intensive Care Units (ICUs) in Egypt. A total of 94 patients participated in this retrospective observational study. Patient’s medical records were used to collect demographics, medical history, admission and discharge dates and medications used. The mean ± SD of the Glasgow Coma Scale (GCS) scores was 9.9 ± 4.4 and the median length of stay was 7 days (range 1–47 days). The total number of prescribed medications ranged from 4–29 with a mean ± SD of 14.1 ± 5.5 medications per patient. The top three most prescribed categories belonged to (1) anti-infective agents (23.9%); (2) electrolyte, caloric and water balance agents (14.6%); and (3) blood formation, coagulation and thrombosis (11.3%). The proton pump inhibitor, esomeprazole, was the most frequently prescribed medication accounting for 6.5% of total prescriptions, followed by clindamycin and magnesium sulfate each accounting for 3.5% of total prescriptions. The potential Drug-Drug Interactions (pDDIs) showed a total of 968 pDDIs with a mean ± SD (range) of 10.2 ± 9.4 (0–43) pDDIs per patient: severe (contraindicated) (3), major (178), moderate (618) and minor (169). Overall, the drug utilization patterns in this study were consistent with ICU drug utilization from other countries in the region. The implementation of clinical decision support systems and the involvement of clinical pharmacists may help improve medication safety.

## 1. Introduction

The examination of drug utilization patterns within intensive care units (ICUs) is critical to identify trends or associations of medication errors with prescription patterns. Medication errors are common and account for 78% of serious medical errors in the ICU [1]. ICUs deal with the most critically ill patients which are typically prescribed twice as many medications as patients in other hospital wards and are most likely to suffer a potentially life threating error at some point during their ICU stay [2]. Medication reconciliation is recommended to improve patient safety [3]. That would include maintaining a list of long-standing medications (reasons for holding or discontinuing) as well as information related to starting new medications and their planned stop dates [3]. The authors also recommended involving pharmacists in patient rounds in the ICU as a means of decreasing adverse drug events [3].

There are few reports on drug utilization in intensive care units in Egypt [4]. To our knowledge, this is the first report of drug utilization and drug-drug interactions at the ICU of a university tertiary care hospital in Egypt. Thus, the main objectives of this retrospective observational study were to assess the drug utilization patterns and potential drug-drug interactions in the ICU at Ain Shams University Internal Medical Hospital, Cairo, Egypt.

## 2. Materials and Methods

### 2.1. Clinical Setting

Ain Shams University’s Faculty of Medicine is a public Egyptian State School and one of the largest educational medical institutions in Africa and the Middle East [5]. The university educational hospitals consist of inpatient and outpatient clinics with approximately 3200 beds and a staff of 10,000 serving approximately 2 million patients per year from all over Egypt. Specialized intensive care units serve individual medical departments (Ain Shams University—Faculty of Medicine). The study was conducted at the medical ICU of Ain Shams University Internal Medicine hospital, Cairo, Egypt. The medical ICU at the hospital has 17 beds and admits adult patients from the internal medicine and oncology departments.

### 2.2. Data Collection and Analysis

A total of 94 patients were admitted to a 17-bed medical ICU at Ain Shams University Internal Medical Hospital, Cairo, Egypt. The patient’s records were used to retrospectively collect all required data for the study. The patients had peripheral or central venous access according to their clinical condition as well as nasogastric tubes used for enteral feeding and the delivery of oral medications. All the patients were admitted between 1 October and 30 November 2018. Data were collected from the clinical pharmacy department and nursing medication administration records. The data included the patients’ demographics (age, weight, gender and ethnicity), past medical history, cause of admission, admission and discharge dates, length of stay, Glasgow Coma Scale (GCS), creatinine clearance, patient outcome and medication administration record. Information on medications prescribed to each patient at the time of admission to ICU and during the entire duration of the ICU stay was recorded providing a complete medication profile. Medication start and end dates were recorded as well as route of administration and estimated total daily dose. The collected data were entered into an excel worksheet. Administered medications were classified into categories and subcategories according to the American Hospital Formulary Service (AHFS drug information). The potential for drug-drug interactions (pDDIs) for each drug included in patient prescription drug care plans was analyzed with clinical pharmacology^®^ software (ELSEVIER North America, New York, NY 10169, USA, Clinical Decision Support). The software produced severity rankings (severe, major, moderate and minor) for each drug combination included in the care plans. The program reported the potential interaction of two or more other drugs included in the patient care plans. Multi-drug interactions were classified according to the severity of each, and the total number of pDDIs per patient was determined (drug interaction report) [6]. Only descriptive statistics were used to summarize all data collected in the study.

## 3. Results

During the study period, 94 patients were admitted to the ICU with a median length of stay of 7 days (range 1–47 days). The most common causes for admission were altered levels of consciousness or respiratory distress. The most common co-morbidities were hypertension and diabetes. Patient diagnoses included pneumonia, respiratory failure, status epilepticus, cerebral vascular strokes, diabetic ketoacidosis, autoimmune disease exacerbation, hepatic and/or uremic encephalopathy, shock, post cardiac arrest conditions and other causes of admission. Table 1 describes the patient demographics, GCS and creatinine clearance.

**Figure 1:**
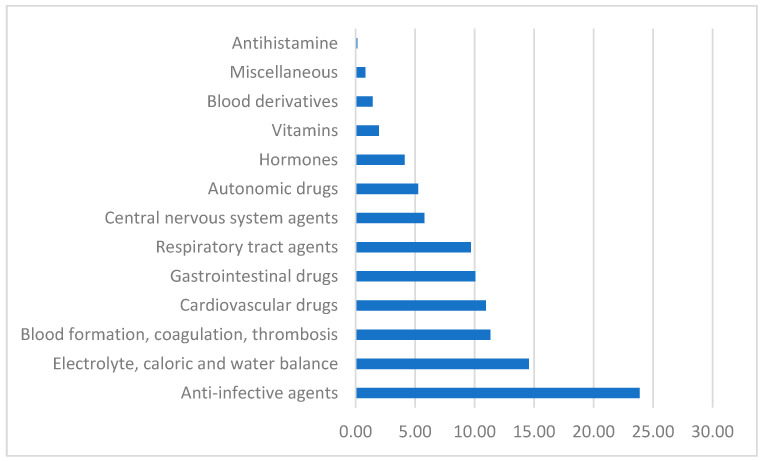
Therapeutic categories of ICU medication utilization (% total prescriptions).

There were more females (62.8%) than males (37.2%) occupying the ICU beds. All patients were of white ethnicity and most of the patients (84.2%) were 41–65 and >66 years of age. Based on estimated creatinine clearance values, most patients had some degree of renal insufficiency with only 13.8% having normal renal function. The total number of medications prescribed for every patient ranged from 4 to 29 with a mean ± SD of 14.1 ± 5.5 medications per patient. The average number of medications prescribed in the first 24 h of stay was nine medications per patient and this number declined to seven medications per patient on day 2 and was variable up to the end of the hospital stay. As would be expected in an ICU, most medications were administered intravenously (62.6%). The oral route accounted for 26% of medications which were mostly anti-hypertensives, laxatives and anti-epileptics. Other routes of administration included subcutaneous injection (8.3%), mostly for DVT prophylaxis and long-acting insulin, and the inhalation route accounted for 3.1% of medications. The overall ICU medication utilization (% of total prescriptions) was grouped according to AHFS into 13 therapeutic categories as displayed in Figure 1.

**Figure 2:**
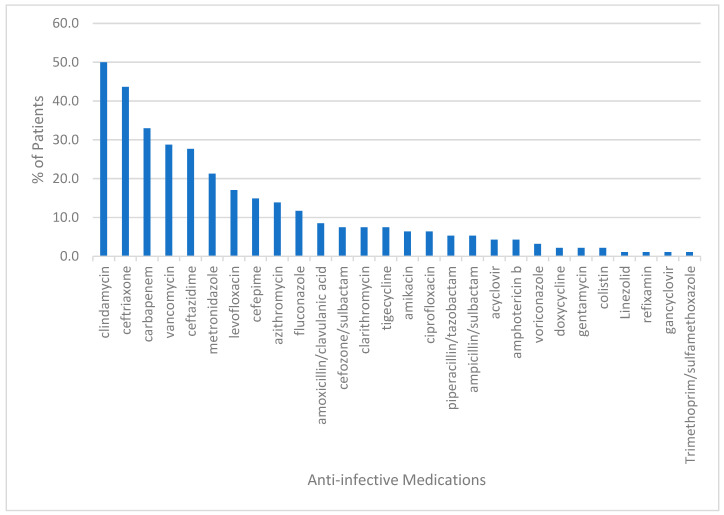
Percent of patients receiving anti-infective agents.

The top three most prescribed medication categories (% of total ICU prescriptions) belonged to the following therapeutic categories: (1) anti-infective agents (23.9%); (2) electrolyte, caloric and water balance agents (14.6%); and (3) blood formation, coagulation and thrombosis (11.3%). The CNS agents included drugs such as paracetamol, midazolam, phenytoin and piracetam. The miscellaneous category contained mostly the antigout agent, allopurinol. Medications belonging to the anti-infective agents were by far the most utilized at the ICU, with clindamycin, ceftriaxone and carbapenem antibiotics accounting for the top antibiotics received by patients (Figure 2).

**Figure 3:**
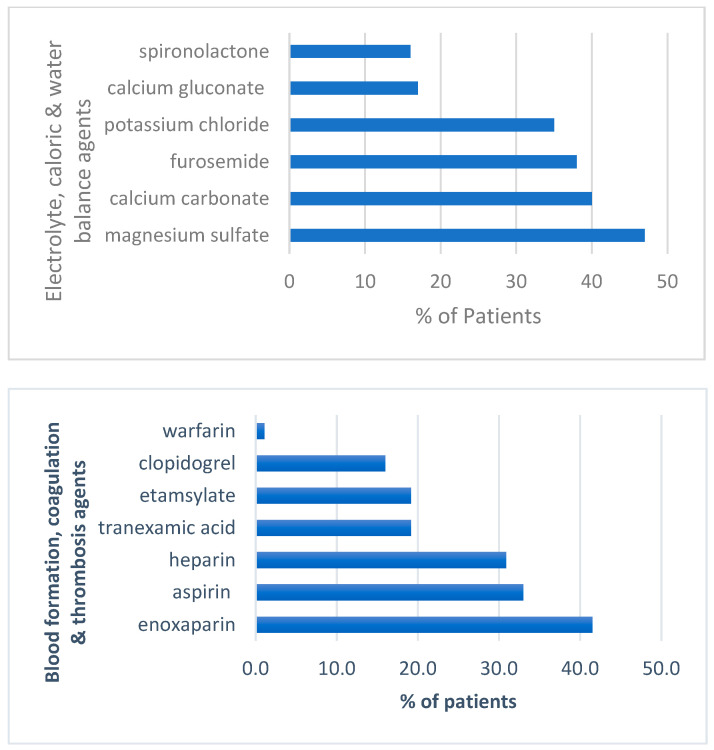
Percent of patients receiving drug agents according to drug categories.

Among all the anti-infective agents, clindamycin was the most prescribed drug, followed by ceftriaxone, which is in agreement with the ICU empirical antibiotic protocol for aspiration pneumonia and infected pressure ulcers. The carbapenem antibiotics, imipenem and meropenem, were only prescribed for ESBL and MDR bacterial infections. In the electrolyte, caloric and water balance agent’s category, magnesium sulfate, calcium carbonate and furosemide were the top agents in that category (Figure 3).

Magnesium sulfate had the highest number of prescriptions in that class of agents as it was used as a bronchodilator in patients with chest infections to relax bronchial muscles and expand the airways allowing more airflow in and out of the lungs [7,8,9]. The large number of prescriptions for calcium carbonate reflected the need to treat hyperphosphatemia and normalize phosphate concentrations in patients with CKD which constituted most ICU patients. The third highest number of prescriptions in this class was for furosemide which is the only intravenous diuretic in the ICU formulary. For the blood formation, coagulation and thrombosis category, enoxaparin was the most prescribed anticoagulant and aspirin was the most prescribed antiplatelet (Figure 3). Enoxaparin was the most used medication for DVT prophylaxis. The 25 most frequently prescribed medications are represented in Table 2.

**Figure 4:**
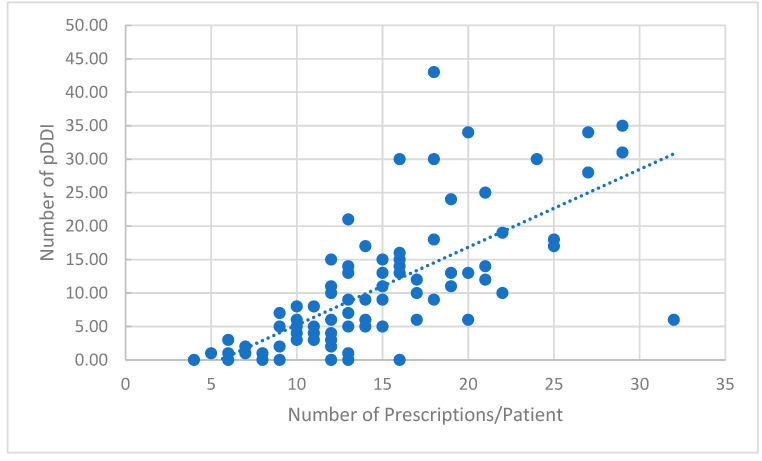
Correlation between the number of potential DDIs and the number of prescriptions per patient (trend line).

The proton pump inhibitor, esomeprazole, was the highest on the list accounting for 6.5% of total prescriptions, followed by clindamycin and magnesium sulfate each accounting for 3.5% of total prescriptions. The pDDIs as evaluated by the Clinical Pharmacology^®^ drug interaction report showed a total of 968 pDDIs with a mean ± SD (range) of 10.2 ± 9.4 (0–43) pDDIs per patient. The breakout of the number of pDDIs was as follows: severe (contraindicated) (3), major (178), moderate (618) and minor (169). During their ICU stay, 87 patients (93%) experienced at least one pDDI. There were no clinically significant negative outcomes that could be directly attributed to the pDDIs. However, it should be noted that the complex pharmacotherapy coupled with patient comorbidities in the ICU setting makes it difficult to diagnose pDDIs in critically ill patients. The three cases of severe pDDIs were for the same drug combination of granisetron and fluconazole. As would be expected, the propensity of potential DDIs increased as a function of the number of prescribed drug agents per patient (Figure 4).

## 4. Discussion

Recent data show that pharmacists can play a key role in medication management in the ICU [10]. Within the critical care professional team, they can intercept and resolve medication errors and optimize medication therapy based on pharmacokinetic and pharmacodynamic principles [10]. The ICU in this study typically receives patients who are in the late stage of disease and require many drug interventions. In the current study, even though there are prescription protocols and/or guidelines for proper drug use in ICUs, sometimes it may not be possible to strictly adhere to them because of a patient’s specific disease state, the complex ICU environment, the rotation of ICU medical residents and the prescribing physician’s medication preferences. Prescribed medication care plans are both therapeutic to treat diagnosed conditions as well as prophylactic to prevent ICU stay associated complications (DVT prophylaxis, antibiotics, antiulcer medications and sedatives). Out of the total 91 medications used, 73 (80%) belonged to the World Health Organization’s (WHO’s) Model List of Essential Medicines [11]. This is consistent with previous studies that showed that about 85–88% of prescribed drugs were from the WHO essential drug list [12]. Patel et al. studied the drug utilization patterns in 397 patients in a critical care unit in India and reported that the mean number of medications used per patient was 13.5 (95% CI 13.05–14.04) which is close to that reported in the present study [13]. In a study of 278 trauma intensive care patients conducted in the US, the mean ± SD number of drugs prescribed was 9.1 ± 6.5 [14]. Only one patient was prescribed warfarin which we typically avoid in the ICU because of its complicated drug interaction profile and the time needed to adjust and stabilize INR values. That time delay does not allow the medical staff to perform any interventional procedures such as central line insertion, tracheostomy, or bedsore debridement. Heparin and LMWH are preferred for patients who are warfarin candidates [15]. For the cardiovascular class, atorvastatin was the most used medication. In addition to its proven cholesterol lowering effect, it is recommended for patients with coronary artery disease to prevent heart failure [16]. For respiratory medications, N-acetylcysteine, a mucolytic and antioxidant drug [17], was used widely to ease mucous suction from intubated patients [18]. For pain and fever control, paracetamol was the most prescribed medication because of the perception of prescribers that it is safer in comparison with NSAIDs, especially in patients who have kidney disease or are prone to gastrointestinal bleeding. Since the ICU was medical non-surgical, the need for opioids for pain management was minimal. Insulin was widely used because the ICU blood sugar control protocol required an insulin infusion pump used or a sliding scale according to each patient’s condition. Norepinephrine was the top of the list of inotropes, and this was consistent with its use according to the ICU sepsis protocol. Data have shown that the use of norepinephrine as part of hemodynamic management may influence outcomes favorably in septic shock patients [19]. In a study of 138 intensive care patients in the country of Oman, omeprazole was the most prescribed drug accounting for 6.7% of all prescriptions which is almost identical to the percentage of esomeprazole reported in the present report [20]. Our study showed that the top prescribed medications belonged to anti-infective agents (23.9% of total ICU prescriptions) with clindamycin being the top anti-infective agent. Data from recent reports indicate that clindamycin significantly reduced mortality in critically ill patients with bacteremic Group A streptococcal infections [21]. Al-Zakwani et al. reported anti-infective drugs were the most prescribed class of drugs accounting for 25.1% which is consistent with our finding of 23.9% for anti-infective drugs as a % of total ICU prescriptions [20]. Gawali and Khobragade reported that anti-infective drugs were the most prescribed drugs (30%) at the ICU of a tertiary care teaching hospital in India [22]. A report on the use of antibiotics in the intensive care unit of a tertiary hospital in Malawi points to even higher use of antibiotics in the ICU. The study showed that 81.6% of patients received antibiotics at the ICU and that bacteria appeared to show high levels of resistance to ceftriaxone, which is the last line antibiotic commonly being used [23]. Another ICU drug utilization study in Oman showed that NSAIDs were the most prescribed class of drugs at 38% [12]. Overall, the drug utilization patterns in the current study were consistent with other reports on ICU drug utilization from other countries in the region.

The identification of pDDIs is challenging in most ICU patients given complexity of drug profiles and the occurrence of adverse drug events [24]. Multidrug interactions are known to increase the risk of associated adverse drug events [25]. A large study of pDDIs in 1659 intensive care patients showed that 54% of patients experienced at least one pDDI [26] In the ICU setting, vast majority of pDDIs can be managed by monitoring [26]. It should be noted that the drug-drug interaction software is designed to flag potential DDIs and does not address clinical significance. Since polypharmacy is unavoidable in the ICU setting, there is the possibility that the combination of drug agents can modify the proarrhythmic potential of one of the drug agents triggering a QT prolongation effect [27]. Because ICU patients are continuously monitored, the offending drug can be stopped if any ECG changes occur. The major pDDIs mostly consist of metabolic type interactions arising from using combinations of a potent inhibitor and substrate drug agents of the same cytochrome P450 family of enzymes. Examples of potent inhibitor perpetrators of pDDIs are clarithromycin and fluconazole (CYP3A). Many drug agents that were widely used in the ICU in this study were inhibitors of important CYP enzymes, i.e., esomeprazole (a competitive inhibitor of CYP2C19), ciprofloxacin (a moderately potent inhibitor of CYP1A2), and fluconazole (an inhibitor of CYP3A4, CYP2C9 and CYP2C19) [28]. Other major pDDIs involved the combination of potassium chloride with potassium sparing diuretics or ACE inhibitors. Major pDDIs involving digoxin were due to its narrow therapeutic index. Due to lack of availability of IV antihypertensive medications at the ICU, multiple oral antihypertensive agents had to be administered via NG tubing in addition to IV nitroglycerin, and that accounted for several moderate pDDIs. The use of aspirin, heparin or any of its derivatives for DVT prophylaxis also accounted for several of the observed pDDIs. A few of the moderate pDDIs resulted from the interaction of insulin with several medications such as steroids, inotropes and beta blockers. Given that 46.8% of ICU patients were diabetic and using insulin, this may have contributed to the number of pDDIs. The frequency of 10.2 pDDIs per patient reported in the current study is much higher than the 1.7 pDDIs per patient reported by Uijtendaal et al. [26], the 2.2 pDDIs per patient reported by Bakker et al. [29] and the 3 pDDIs per patient reported by Vanham et al. [24]. It has been reported that at least one relevant pDDI occurs in 54% of all ICU patients [26]. The percentage of ICU patients experiencing at least one pDDI in the current study was 93% which is higher than those reported in other ICU studies (79% reported by Vanham et al. [24] and 76% reported by Wagh et al. [30]). One of the reasons for the higher pDDIs, as stated earlier, might be that the ICU deals with patients who are in later stages of disease requiring many drug interventions. The introduction of clinical decision support systems (CDSSs) has shown potential in preventing pDDIs [31]. The CDSSs, which takes into consideration clinical knowledge and patient-related information, generates a computerized alert based on clinically relevant pDDIs [32]. Studies in critically ill patients in ICU have shown improvements in the management of infections, anticoagulation therapy, sedation and analgesia by implementing critical care pharmacy services and having clinical pharmacists contributing as members of the interdisciplinary ICU team [33]. Bazan et al. recommended optimizing clinical pharmacy services with prescription intervention strategies to reduce morbidity and mortality in critically ill Egyptian patients with renal insufficiency [4]. Thus, the implementation of a CDSS system and having clinical pharmacists contribute to prescription drug care plans as members of the multidisciplinary ICU team may help improve medication safety in ICU patients.

## 5. Conclusions

In the ICU, the most prescribed drugs were the anti-infective agents; electrolyte, caloric and water balance agents; and the blood formation, coagulation and thrombosis agents. Overall, the drug utilization patterns in this study were consistent with other reports on ICU drug utilization from other countries in the region. The frequency of 10.2 pDDIs per patient reported in the current study is higher than those reported in the literature and underlines the importance of involving clinical pharmacists at the ICU in therapeutic planning at the prescribing stage. While the vast majority of interactions can be managed by monitoring in the ICU setting, the implementation of clinical decision support systems and having clinical pharmacists contribute to prescription drug care plans as members of the multidisciplinary ICU team may help improve medication safety and patient outcomes.

## Figures and Tables

**Table 1:** Demographics and clinical parameters of ICU patients (*n* = 94).

Characteristic	
gender	
male	35 (37.2%)
female	59 (62.8%)
age:	
0–20 years	3 (3.19%)
21–40 years	12 (12.8%)
41–65 years	45 (47.9%)
>66 years	34 (36.2%)
weight (kg) mean ± SD	81.5 ± 16.7
GCS mean ± SD	9.9 ± 4.4
creatinine clearance:	
0–30 mL/min	33 (35.2%)
31–60 mL/min	29 (30.8%)
61–90 mL/min	19 (20.2%)
>90 mL/min	13 (13.8%)
median length of stay (range days)	7 (1–47)

**Table 2:** Top 25 most frequently prescribed medications administered to 94 hospitalized ICU patients.

Rank	Drug Name	Number of Prescriptions	% Total Prescriptions
1	esomeprazole	88	6.5
2	clindamycin	47	3.5
3	magnesium sulfate	47	3.5
4	insulin	44	3.3
5	norepinephrine	43	3.2
6	ceftriaxone	41	3.0
7	calcium carbonate	40	3.0
8	enoxaparin	39	2.9
9	furosemide	38	2.8
10	paracetamol	37	2.7
11	atorvastatin	36	2.7
12	potassium chloride	35	2.6
13	acetylcysteine	32	2.4
14	amlodipine	31	2.3
15	aspirin	31	2.3
16	carbapenem	31	2.3
17	methyldopa	31	2.3
18	heparin	29	2.1
19	vancomycin	27	2.0
20	ceftazidime	26	1.9
21	hydrocortisone	25	1.8
22	ipratropium	24	1.8
23	granisetron	22	1.6
24	alfacalcidol	21	1.6
25	metronidazole	20	1.5

## Data Availability

The data sets used and analyzed during the current study are available from the corresponding author on a reasonable request.

## Abbreviations

AHFS	American Hospital Formulary Service
CDSSs	clinical decision support systems
CKD	chronic kidney disease
DVT	deep vein thrombosis
ESBL	extended spectrum beta-lactamase producing bacteria
GCS	Glasgow Coma Scale
ICU	intensive care unit
INR	international normalized ratio
LMWH	low molecular weight heparin
MDR	multidrug-resistant bacteria
NSAIDs	non-steroidal anti-inflammatory drugs
pDDI/s	potential drug-drug interaction/s
WHO	World Health Organization
